# A pilot study on primary cultures of human respiratory tract epithelial cells to predict patients’ responses to H7N9 infection

**DOI:** 10.18632/oncotarget.24537

**Published:** 2018-02-20

**Authors:** Chung-Guei Huang, Li-Ang Lee, Yi-Cheng Wu, Mei-Jen Hsiao, Jim-Tong Horng, Rei-Lin Kuo, Chih-Heng Huang, Ya-Chu Lin, Kuo-Chien Tsao, Min-Chi Chen, Tse-Ching Chen, Shin-Ru Shih

**Affiliations:** ^1^ Research Center for Emerging Viral Infections, Chang Gung University, Taoyuan 33302, Taiwan, ROC; ^2^ Graduate Institute of Biomedical Sciences, Department of Medical Biotechnology and Laboratory Science, College of Medicine, Chang Gung University, Taoyuan 33302, Taiwan, ROC; ^3^ Department of Laboratory Medicine, Linkou Chang Gung Memorial Hospital, Taoyuan 33305, Taiwan, ROC; ^4^ Department of Otorhinolaryngology - Head and Neck Surgery, Linkou Chang Gung Memorial Hospital, Taoyuan 33305, Taiwan, ROC; ^5^ Faculty of Medicine, College of Medicine, Chang Gung University, Taoyuan 33302, Taiwan, ROC; ^6^ Department of Surgery, Linkou Chang Gung Memorial Hospital, Chang Gung University, Taoyuan 33305, Taiwan, ROC; ^7^ Graduate Institute of Biomedical Sciences, Department of Biochemistry and Molecular Biology, College of Medicine, Chang Gung University, Taoyuan 33302, Taiwan, ROC; ^8^ Institute of Preventive Medicine, National Defense Medical Center, Taipei 11490, Taiwan, ROC; ^9^ Department of Public Health and Biostatistics Consulting Center, Chang Gung University, Taoyuan 33302, Taiwan, ROC; ^10^ Department of Pathology, Linkou Chang Gung Memorial Hospital, Chang Gung University, Taoyuan 33305, Taiwan, ROC

**Keywords:** avian influenza A(H7N9) virus, primary epithelial cells, tropism, cytokine, patient-related characteristics

## Abstract

Avian influenza A(H7N9) virus infections frequently lead to acute respiratory distress syndrome and death in humans. We aimed to investigate whether primary cultures of human respiratory tract epithelial cells are helpful to understand H7N9 virus pathogenesis and tissue tropism, and to evaluate how patient-related characteristics can affect the host's response to infection. Normal human bronchial epithelial cells (isolated from two different donors) and primary epithelial cells (harvested from 27 patients undergoing airway surgery) were experimentally infected with H7N9 and/or H1N1pdm for 72 h. After virus infection, the culture media were collected for viral RNA quantitation and cytokine detection. Both H7N9 and H1N1pdm viruses replicated and induced a cytokine response differently for each donor in the normal human bronchial epithelial model. H7N9 replicated equivalently in epithelial cells harvested from the inferior turbinate and paranasal sinus, and those from the larynx and bronchus, at 72 h post-infection. Viral RNA quantity at 72 h was significantly higher in patients aged 21–64 years than in patients aged ≥ 65 years; however, no effects of sex, medical comorbidities, and obesity were noted. H7N9-infected cultured cells released multiple cytokines within 72 h. Levels of interleukin-1β, interleukin-6, interleukin-8, interferon-γ, and tumor necrosis factor-α were associated differently with patient-related characteristics (such as age, sex, obesity, and medical comorbidities). In the era of precision medicine, these findings illustrate the potential utility of this primary culture approach to predict a host's response to H7N9 infection or to future infection by newly emerging viral infections, and to dissect viral pathogenesis.

## INTRODUCTION

Avian influenza A(H7N9) virus infections are a serious public health threat. H7N9 infections were initially reported in China in March 2013 [1]. The clinical presentation of H7N9 infections varies with the individual; however, fever and cough generally represent the core symptoms [2]. Serious complications include pneumonia, acute respiratory distress syndrome, and death. Although the first outbreak has subsided, four subsequent seasonal epidemics were observed in China [3]. The severe illness of H7N9 infections and the presence of natural reservoirs represent significant concerns. In this context, the identification of vulnerable subjects is paramount to the prevention of spread.

H7N9 transmission generally occurs from poultry to humans, and the closure of live poultry markets has been an effective control strategy [4]. However, evidence also indicates that: 1) H7N9 viruses are transmissible from ferrets in a direct contact setting [5–7]; 2) they can bind to both human and avian receptors [8]; 3) they can attach to the epithelium in both the upper and lower respiratory tracts of humans [9]; and 4) they efficiently propagate in human alveolar tissue [10]. All of these features are important factors for human-to-human transmission. Fortunately, H7N9 viruses do not appear to transmit easily from person to person. However, there are many more factors that are important in the adaptation to sustained inter-human transmission. In this scenario, primary cultures of human respiratory tract epithelial cells would be invaluable to understand H7N9 virus tissue tropism and pathogenesis, as well as to evaluate how patient-related characteristics can modulate the host's response to infection.

Notably, old age (≥ 65 years), male sex, medical comorbidities, and obesity have been previously identified as risk factors for the development of severe disease [2, 11–15]. An age- and sex-based analysis of 63 patients infected by the H7N9 virus showed that elderly men are most commonly affected, whereas no deaths were observed in elderly women. Age-dependent changes in the airway epithelium mean that older patients may be more prone to respiratory tract infections [16] and the development of symptomatic H7N9 disease [11]. Moreover, the presence of comorbidities is the only independent risk factor for acute respiratory distress syndrome in H7N9-infected individuals [2]. In the most recent epidemic episode in China (during September to December 2016 (*n* = 114)), distributions of age, sex, and severe illness remained unchanged compared with other epidemics [3]. Nevertheless, the mechanisms by which age, sex, medical comorbidity, and obesity can influence H7N9 virus replication kinetics remain unclear. However, numerous studies have shown that increased levels of specific proinflammatory cytokines and chemokines are robust predictors of morbidity and mortality in H7N9-infected patients [2, 8, 17–20].

H1N1pdm virus is a swine-origin influenza virus that can transmit efficiently among humans. Although the clinical severity of H1N1pdm was milder than that of avian H7N9 virus, there were many severe H1N1pdm patients because of the widespread transmission of the virus in the human population in 2009 [21]. Compared with H7N9 virus infections, young age (≤ 29 years) and female sex were identified as risk factors for severe H1N1pdm infection [15].

Respiratory epithelial cell culture systems have been used to investigate the interaction between influenza viruses, as well as other viruses, and their host, [8, 22–25]. While these studies have recognized many important characteristics that can help to understand the pathogenesis of influenza viruses, few studies have investigated person-to-person differences in virus replication and the influenza-related cytokines and chemokines of respiratory epithelial cell cultures. Recently, Mindaye *et al.* [26] found different protein expression levels of pro-viral and antiviral factors among normal human bronchial epithelial (NHBE) cells isolated from three different donors, and suggested that this model may provide a way to identify individuals or population groups who are susceptible to severe influenza disease. In the present study, we addressed the hypothesis that respiratory epithelial cells from different human donors would vary in their response to influenza virus infections. To determine the impact of age on cellular response after influenza virus infection, commercial NHBE cells cultured from 24-year-old and 69-year-old donors were infected with both H1N1pdm virus (A/California/07/2009 [27]) and avian H7N9 virus (A/Taiwan/4-CGMH/2014 [28]), and viral growth kinetics and the cytokine response were compared. We further explored how different donors’ characteristics (i.e., age, sex, medical comorbidities, and obesity) could influence both virus replication kinetics and the cytokine response to experimental H7N9 infections. Human respiratory tract primary epithelial cells were cultured from patients undergoing upper or lower airway surgery and experimentally infected with H7N9. The limited amount of primary epithelial cells available from each patient meant that only H7N9 virus infection was conducted.

## RESULTS

### Comparison of the H7N9 and H1N1pdm viral RNA quantities in NHBE cells from different donors

**Figure 1:**
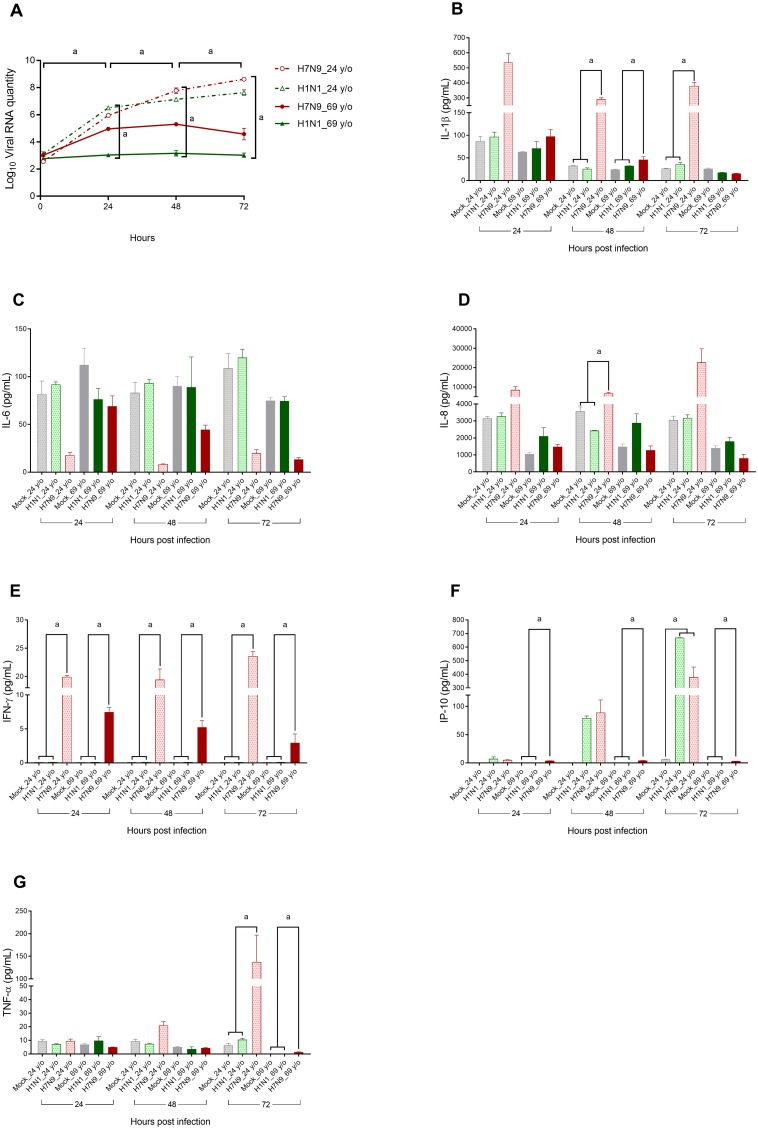
Changes in viral RNA quantity (panel **A**) and core cytokine levels (panels **B**–**G**) in culture supernatants of normal human bronchial epithelial cell cultures in relation to variables of interest. Normal human bronchial epithelial cells were cultured from a 24-year-old female and a 69-year-old female and experimentally infected with the H7N9 or H1N1pdm virus. Viral RNA quantity (log-transformed) and cytokine values were compared using the Mann–Whitney *U* test or Kruskal–Wallis test to evaluate their differences at each time point, or a Wilcoxon signed rank test to evaluate their difference between various time points. ^a^*P* < 0.05.

In consideration of biosafety issues, the viral RNA quantity was determined to represent changes in viral titer at each time point. Figure 1A demonstrates that the viral RNA quantity increased significantly from 1 to 72 h post infection (p.i.) in the culture supernatants of the NHBE cells after experimental infection with the H7N9 virus or H1N1pdm virus, when all cultures were considered in combination. In the H1N1pdm virus or H7N9 virus-infected supernatants, viral RNA quantity reached a plateau between 48 and 72 h p.i. (*P* = 0.753 and 0.249, respectively). Table 1 summarizes the viral RNA quantity in influenza virus-infected culture supernatants harvested from NHBE cells isolated from two different donors. Differences in viral RNA quantities between H7N9 and H1N1pdm virus infections were not significant at each time point in the same NHBE cells. Viral RNA quantities of both influenza viruses measured in culture medium of NHBE cells obtained from a 24-year-old female donor were significantly higher than those obtained from a 69-year-old donor at each time point (*P* = 0.041, 0.002, 0.002, and 0.002, respectively). Furthermore, the viral RNA quantity detected in H7N9-infected cell culture medium of the 24-year-old NHBE cells was the highest among all cultures from 48 to 72 h p.i., whereas the viral RNA quantity of the H1N1pdm-infected 69-year-old culture medium was the lowest from 24 to 72 h p.i. (all *P* values < 0.05).

**Table 1:** Viral RNA quantity and cytokine levels in H1N1pdm virus and H7N9 virus-infected culture supernatants harvested from normal human bronchial epithelial cells isolated from two different donors

	24-year-old female							69-year-old female						
	Mock		H1N1pdm		H9N9		*P*	Mock		H1N1pdm		H9N9		*P*
	Mean	SEM	Mean	SEM	Mean	SEM		Mean	SEM	Mean	SEM	Mean	SEM	
Viral RNA quantity (log_10_ transformation)														
1 h p.i,	–	–	3.1	0.17	2.55	0.15	0.200	–	–	2.77	0.11	3.02	0.09	0.100
24 h p.i.	–	–	6.51	0.12	5.95	0.08	0.100	–	–	3.04	0.09	4.96	0.11	0.100
48 h p.i.	–	–	7.14	0.13	7.78	0.2	0.100	–	–	3.16	0.21	5.3	0.1	0.100
72 h p.i.	–	–	7.64	0.2	8.62	0.08	0.10	–	–	3.02	0.16	4.58	0.43	0.700
IL-1β (pg/mL)														
24 h p.i.	86.33	11.36	96.15	10.8	534.48	60.75	0.061	62.85	1.82	70.77	15.64	97.05	15.37	0.148
48 h p.i.	31.95	1.28	25.33	2.62	289.02	13.13	0.039	24	1.2	32.02	0.67	45.51	7.11	0.027
72 h p.i.	26.24	1.02	35.52	4.21	379.3	24.76	0.039	25.46	2.2	17	1.54	15.21	0.62	0.051
IL-6 (pg/mL)														
24 h p.i.	81.19	14.36	91.6	2.97	17.64	3.19	0.061	112.03	17.82	76.15	11.62	68.8	11.19	0.288
48 h p.i.	82.87	11.28	93.1	3.79	8.16	0.55	0.061	90.07	10.2	88.83	31.91	44.41	4.84	0.099
72 h p.i.	108.42	16.02	119.69	8.8	19.46	4.1	0.051	74.7	3.2	74.36	4.72	13.15	2.09	0.066
IL-8 (pg/mL)														
24 h p.i.	3146.68	116.36	3255.66	216.57	8444.22	1814.32	0.061	1038.79	102.33	2094.51	510.89	1473.57	144.2	0.051
48 h p.i.	3549.17	280.28	2416.11	29.04	6711.78	646.5	0.027	1464.8	181.03	2870.38	549.54	1267.79	257.94	0.061
72 h p.i.	3023.71	260.22	3156.84	203.76	22563.5	7183.65	0.059	1387.82	152.66	1788.49	245.31	783.81	246.22	0.079
IFN-γ (pg/mL)														
24 h p.i.	0.1	0	0.1	0	19.9	0.31	0.021	0.1	0	0.1	0	7.45	0.7	0.022
48 h p.i.	0.1	0	0.1	0	19.39	1.95	0.022	0.1	0	0.1	0	5.22	1.02	0.021
72 h p.i.	0.1	0	0.1	0	23.57	0.84	0.022	0.1	0	0.1	0	2.92	1.33	0.022
IP-10 (pg/mL)														
24 h p.i.	0.1	0	6.88	3.89	4.95	0.54	0.126	0.1	0	0.1	0	3.61	0.20	0.022
48 h p.i.	0.1	0	78.64	4.42	88.43	22.87	0.055	0.1	0	0.1	0	3.84	0.53	0.022
72 h p.i.	6.07	0.63	668.35	1.65	377.47	76.11	0.027	0.1	0	0.1	0	3.01	0.13	0.021
TNF-α (pg/mL)														
24 h p.i.	9.19	1.4	6.87	0.64	9.41	1.55	0.301	6.8	0.9	9.67	3.07	4.84	0.27	0.430
48 h p.i.	9.19	1.66	7.23	0.53	20.86	2.87	0.051	5.01	0.51	3.44	1.8	4.21	0.7	0.733
72 h p.i.	6.26	1.52	10.33	0.98	136.81	59.94	0.039	0.1	0	0.1	0	1.19	0.44	0.022

Abbreviations: IFN, interferon; IL, interleukin; IP-10, IFN-γ-induced protein-10; p.i., post infection; TNF, tumor necrosis factor.

All values were compared using the Mann–Whitney U test or Kruskal–Wallis test, as appropriate.

### Comparison of the H7N9 and H1N1pdm cytokine responses in NHBE cells from different donors

Influenza virus infection can induce a cytokine storm, and tumor necrosis factor (TNF), interferon (IFN), interleukin (IL)-1, IL-6, and monocyte chemotactic protein-1 are key cytokine storm mediators [29]. An early-onset cytokine storm is associated with H7N9-related mortality [18, 20]; therefore, we specifically focused on the six cytokines that were found to most consistently predict death in previous studies (i.e., IL-1β, IL-6, IL-8, IFN-γ, interferon gamma-induced protein 10 (IP-10), and TNF-α). Such molecules are also produced by human airway epithelial cells [30–34], and are termed “core cytokines” in the present study. In cases in which the cytokine concentration was undetectable, we assumed a value of 0.1 pg/mL for statistical purposes, in accordance with the recommendation of Guo et al. [20]. Figure 1B shows that the IL-1β levels in the culture media of H7N9 virus-infected 24-year-old NHBE cells were significantly higher than those of the Mock and H1N1pdm virus-infected 24-year-old NHBE cells at 48 and 72 h p.i. The IL-1β levels of the H7N9 virus-infected 69-year-old NHBE cells were significantly higher than those of Mock and H1N1pdm virus-infected 69-year-old NHBE cells at 48 h p.i. Figure 1C reveals that the IL-6 levels in the culture media were not related to virus type or the donor's age. In Figure 1D, the IL-8 level of the culture medium of H7N9-infected 24-year-old NHBE cells was significantly higher than those of the Mock and H1N1pdm infected cells. Figure 1E shows that the IFN-γ level of H7N9-infected NHBE cells (either 24-year-old or 69-year-old) was significantly higher than those of the Mock and H1N1pdm infected cells. Figure 1F demonstrates that levels of IP-10 in H7N9-infected 69-year-old NHBE cells were significantly higher than the others at 24, 48, and 72 h p.i., whereas those of H7N9 and H1N1pdm virus-infected 24-year-old NHBE culture media were significantly higher than that of Mock at 72 h p.i. Figure 1G demonstrates that the levels of TNF-α of H7N9 virus-infected NHBE cells obtained from 24-year-old and 69-year-old donors were significantly higher than the others at 72 h p.i.

### Patients and tissue specimens

Airway tissue specimens were collected from 27 patients (Table 2). There were 19 males (70%) and eight females (30%), with a mean age of 45.6 ± 15.7 years. Patients were further dichotomized into “aged 21–64 years (*n* = 23 [85%])” and “aged ≥ 65 years (*n* = 4 [15%])” groups. Seven (26%) patients had at least one medical comorbidity (hypertension [*n* = 6], diabetes mellitus [*n* = 2] and asthma [*n* = 2]); and nine (33%) were obese (body mass index ≥ 27 kg/m^2^ [35]). The anatomical sites from which the primary epithelial cell cultures were obtained were as follows: inferior turbinate (*n* = 14 [52%]), paranasal sinus (*n* = 4 [15%]), larynx (*n* = 1 [4%]), and bronchus (*n* = 8 [30%]). There were relatively fewer numbers of paranasal sinus- and larynx-derived cultures; therefore, we combined the inferior turbinate and paranasal sinus-derived cultures as “upper anatomical location” (*n* = 18 [67%]) and larynx- and bronchus-derived cultures as “lower anatomical location” (*n* = 9 [33%]) for statistical evaluation. Spearman's rank correlation coefficients revealed that a lower anatomical location was significantly inversely associated with male sex (*r* = −0.75, *P* < 0.001). Age ≥ 65 years, medical comorbidity, and obesity were not significantly associated with the other patient characteristics (all *P* > 0.05). The epithelial origin (≥ 95%) of cultured cells was confirmed by immunofluorescence staining using an anti-cytokeratin 19 antibody.

**Table 2:** Characteristics of patients from whom respiratory tract epithelial cells were obtained

No.	Site of explant	Age, years	Sex	Medical comorbidities	Obesity
1	Paranasal sinus	24	Male	None	No
2	Inferior turbinate	48	Male	HT	Yes
3	Paranasal sinus	74	Male	None	No
4	Inferior turbinate	39	Male	None	Yes
5	Larynx	22	Female	None	Yes
6	Inferior turbinate	38	Male	None	No
7	Inferior turbinate	32	Male	None	No
8	Inferior turbinate	38	Male	DM	Yes
9	Paranasal sinus	38	Male	DM, HT, asthma	Yes
10	Bronchus	47	Female	None	No
11	Paranasal sinus	56	Male	None	No
12	Inferior turbinate	49	Male	None	No
13	Bronchus	63	Female	None	No
14	Paranasal sinus	44	Male	HT	No
15	Inferior turbinate	24	Male	None	No
16	Bronchus	62	Female	None	Yes
17	Paranasal sinus	26	Female	None	No
18	Bronchus	43	Female	None	No
19	Paranasal sinus	70	Male	None	No
20	Inferior turbinate	34	Male	HT	No
21	Inferior turbinate	41	Male	None	No
22	Bronchus	61	Male	None	No
23	Paranasal sinus	38	Male	None	No
24	Inferior turbinate	35	Male	None	Yes
25	Bronchus	66	Female	None	Yes
26	Paranasal sinus	78	Female	HT	No
27	Inferior turbinate	40	Male	HT, asthma	Yes

Abbreviations: DM, diabetes mellitus; HT, hypertension.

### Viral RNA quantitation in human respiratory tract primary epithelial cells

**Figure 2:**
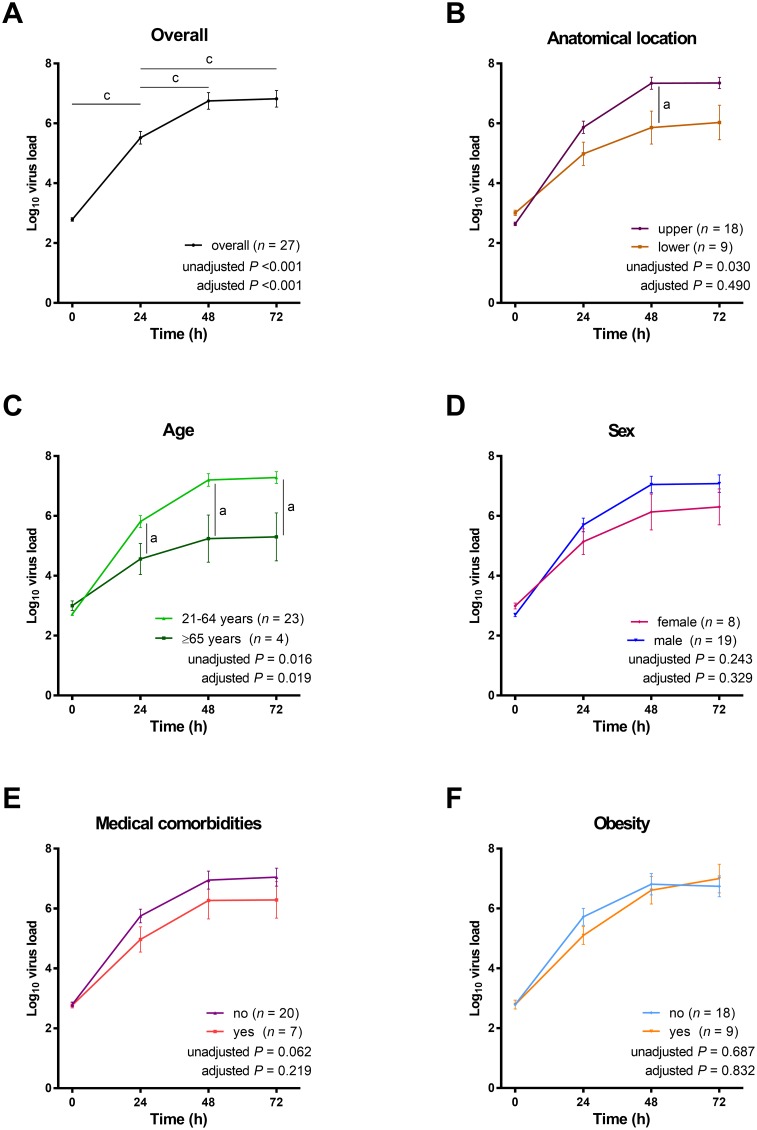
Changes in H7N9 viral RNA quantities in primary cultures in relation to variables of interest All values were log-transformed and compared using generalized estimating equations in which age, sex, medical comorbidity, obesity, and/or anatomical location were included as confounding variables. When all cultures were considered, we observed that viral RNA quantities reached a plateau from 48 to 72 h post-infection (panel **A**). The anatomical location of the explant was not significantly associated with the viral RNA quantities (panel **B**), whereas age was significantly related to viral RNA quantities (panel **C**). Moreover, viral RNA quantities were not significantly associated with sex (panel **D**), medical comorbidities (panel **E**), and obesity (panel **F**).^a^*P* < 0.05, ^b^*P* < 0.01, and ^c^*P* < 0.001, Wilcoxon signed rank test (unadjusted) and generalized estimating equations in which age, sex, medical comorbidity, obesity, and/or anatomical location were included as confounding variables (adjusted).

When all cultures were considered in combination, viral RNA quantities increased significantly at both 24 and 48 h p.i. compared with the baseline, followed by a plateau between 48 and 72 h after infection (Figure 2A), as suggested by generalized estimating equations (GEEs), with or without adjustment for anatomical location, age, sex, medical comorbidities, and obesity. GEEs can account for possible correlations in repeated measures over time and are suitable to explore the differences among different time points [36]. The percentage change in viral RNA quantity between 24 and 72 h was also significant (Table 3).

**Table 3:** Changes in viral RNA quantity and cytokine levels in virus-infected culture supernatants between 24 and 72 h post-infection

	24 h post-infection		72 h post-infection		Percentage Change		*P*	FDR
	Mean	SEM	Mean	SEM	Mean	SEM		
Viral RNA quantity	5.76	0.18	7.21	0.19	11363	4256	< 0.001	0.007^a^
IL-1β	0.89	0.13	1.39	0.13	986	301	< 0.001	0.014^a^
IL-6	3.85	0.13	3.89	0.14	420	244	0.011	0.043^a^
IL-8	4.33	0.10	4.54	0.08	105	45	0.029	0.050^a^
IFN-γ	1.76	0.07	2.02	0.09	215	53	< 0.001	0.021^a^
IP-10	2.42	0.22	3.18	0.20	1193	772	< 0.001	0.029^a^
TNF-α	1.46	0.07	1.65	0.06	139	38	0.003	0.036^a^

Abbreviations: FDR, false discovery rate; IFN, interferon; IL, interleukin; IP-10, IFN-γ-induced protein-10; TNF, tumor necrosis factor.

All values were log-transformed (the only exceptions being percentage changes) and compared using the Wilcoxon signed rank test.

^a^ Indicates a statistically significant FDR when the Benjamini and Hochberg approach for multiple testing correction was applied.

### Viral RNA quantity in primary epithelial cultures in relation to the anatomical site of the explant

With regard to virus tropism, viral RNA quantities were significantly higher in epithelial cells obtained from the upper anatomical locations than from the lower anatomical locations, without adjustment (*P* = 0.030); however, the difference lost significance after adjustment for age, sex, medical comorbidities, and obesity (*P* = 0.490; Figure 2B).

### Viral RNA quantity in primary epithelial cultures in relation to patient-related characteristics

Viral RNA quantities in cells explanted from patients aged ≥ 65 years were significantly lower than those measured in patients aged 21–64 years, with or without adjustment for anatomical location, sex, medical comorbidities, and obesity (unadjusted *P* = 0.016, adjusted *P* = 0.019; Figure 2C). In contrast, no significant sex-related differences in viral RNA quantities were evident (Figure 2D). The viral RNA quantities in primary epithelial cells from patients with or without medical comorbidities did not differ significantly (Figure 2E). Similarly, the impact of obesity on viral RNA quantities was not evident (Figure 2F).

### Cytokine levels in virus-infected culture supernatants

The results of the cytokine analysis showed that the concentrations of all six cytokines in virus-infected culture supernatants increased significantly from 24 to 72 h p.i. (Table 3). The largest increase was observed for IP-10. IP-10 mediates both necrotic inflammation [37] and lung injury [38]; therefore, our primary culture model supports the hypothesis that a cytokine storm could mediate airway tissue necrosis during the early stages of H7N9 infection.

### Associations between viral RNA quantities and cytokine levels in virus-infected culture supernatants

Viral RNA quantities were positively correlated with the concentrations of IP-10 and TNF-α at both 24 and 72 h p.i. (Table 4). Notably, the concentrations of IL-6, which were previously reported to be increased in the plasma of H7N9-infected patients during the first week p.i. [20], were not significantly associated with viral RNA quantities using this primary culture model.

**Table 4:** Spearman's correlations between viral RNA quantity and cytokine levels in virus-infected culture supernatants between 24 and 72 h post-infection

Cytokine	Viral RNA quantity at 24 h post-infection			Viral RNA quantity at 72 h post-infection		
	*r*	p	FDR	*r*	p	FDR
IL-1β	- 0.061	0.762	0.050	0.438	0.022	0.025^a^
IL-6	- 0.090	0.656	0.033	0.060	0.765	0.042
IL-8	0.073	0.718	0.042	- 0.034	0.865	0.050
IFN-γ	0.175	0.383	0.025	0.305	0.122	0.033
IP-10	0.625	< 0.001	0.008^a^	0.530	0.004	0.017^a^
TNF-α	0.478	0.012	0.017^a^	0.683	< 0.001	0.008^a^

Abbreviations: FDR, false discovery rate; IFN, interferon; IL, interleukin; IP-10, IFN-γ-induced protein-10; TNF, tumor necrosis factor.

^a^ Indicates a statistically significant FDR when the Benjamini and Hochberg approach for multiple testing correction was applied.

### Associations between increases in viral RNA quantities over time and core cytokine levels in relation to patient-related characteristics

The associations between the observed increases in viral RNA quantities over time (between 24 and 72 h p.i.) and core cytokine levels were then analyzed in relation to patient-related characteristics (Table 5). Obesity was positively correlated with increases in viral RNA quantities, whereas negative associations were observed for IL-1β and IP-10 levels.

**Table 5:** Spearman's correlations between patient-related characteristics, changes in viral RNA quantities, and changes in core cytokine levels in virus-infected culture supernatants between 24 and 72 h post-infection

Changes in Viral RNA Quantities/Cytokine Levels	Age ≥ 65 Years			Male Sex			Medical Comorbidities			Obesity		
	*r*	*P*	FDR	*r*	*P*	FDR	*r*	*P*	FDR	*r*	*P*	FDR
Viral RNA quantity	- 0.127	0.527	0.036	−0.021	0.918	0.050	0.163	0.417	0.007	0.600	0.001	0.007^a^
IL-1β	- 0.080	0.690	0.050	- 0.073	0.718	0.029	- 0.109	0.590	0.029	- 0.454	0.017	0.021^a^
IL-6	0.282	0.154	0.007	0.125	0.533	0.014	- 0.076	0.706	0.036	- 0.223	0.264	0.036
IL-8	0.222	0.267	0.021	0.167	0.405	0.007	- 0.005	0.979	0.050	- 0.238	0.232	0.029
IFN-γ	- 0.147	0.464	0.029	0.052	0.796	0.036	0.119	0.553	0.021	- 0.131	0.514	0.050
IP-10	0.255	0.199	0.014	- 0.052	0.796	0.043	0.044	0.829	0.043	- 0.465	0.014	0.014^a^
TNF-α	0.120	0.549	0.043	−0.094	0.642	0.021	0.152	0.449	0.014	0.152	0.449	0.043

Abbreviations: FDR, false discovery rate; IFN, interferon; IL, interleukin; IP-10, IFN-γ-induced protein; TNF, tumor necrosis factor.

^a^ Indicates a statistically significant FDR when the Benjamini and Hochberg approach for multiple testing correction was applied.

### Changes in core cytokine levels over time in relation to the anatomical sites of the explant and patient-related characteristics

**Figure 3:**
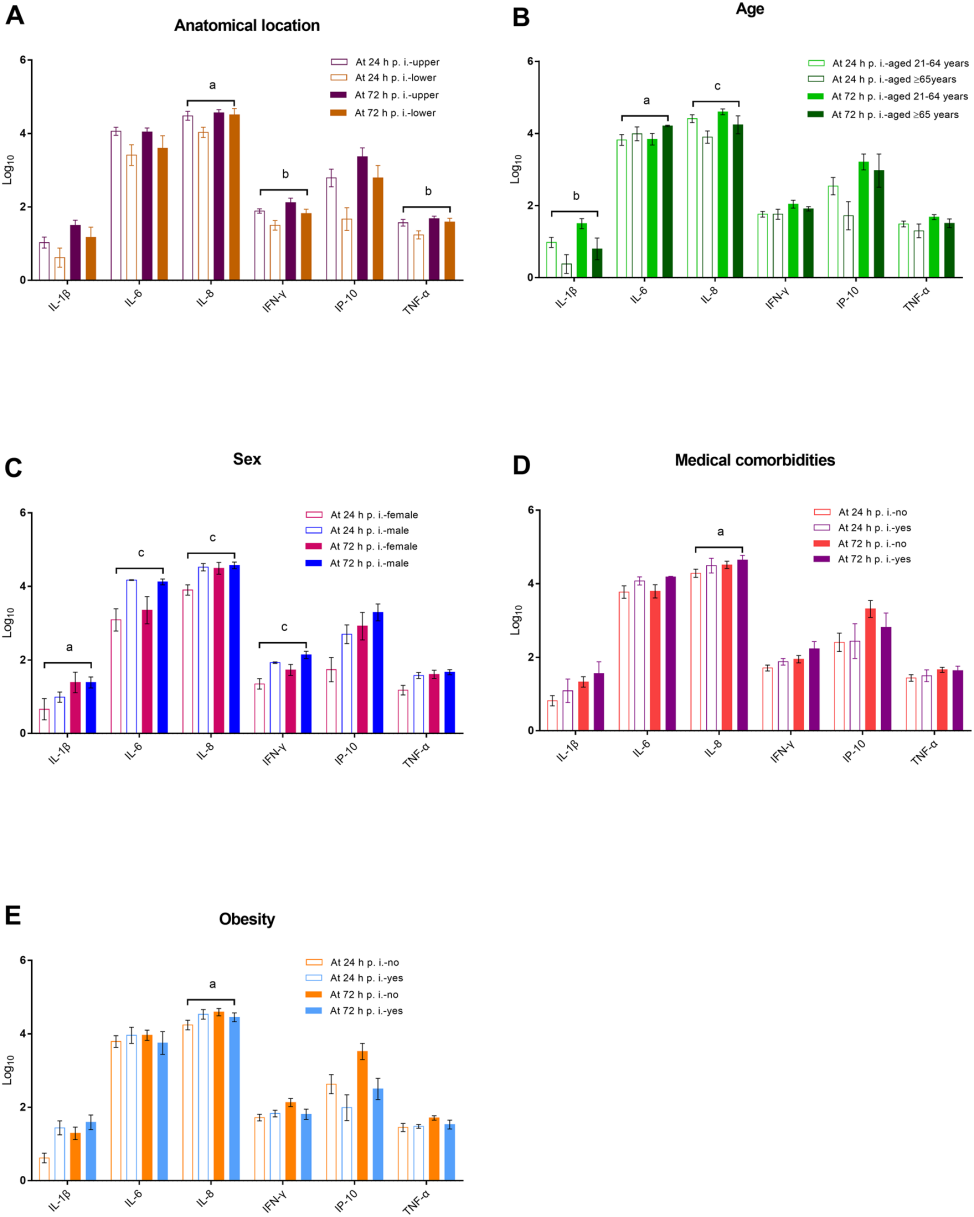
Changes in core cytokine levels in virus-infected culture supernatants in relation to the anatomical sites of the explant and patients’ age, sex, medical comorbidities, and obesity (panels A–E) ^a^*P* < 0.05, ^b^*P* < 0.01, and ^c^*P* < 0.001, generalized estimating equations in which age, sex, medical comorbidity, obesity, and/or anatomical location were included as confounding variables.

Finally, we graphically summarized the changes in core cytokine levels over time in relation to the anatomical sites of the explant and patient-related characteristics. At both 24 and 72 h p.i., the levels of IL-8 and IFN-γ in virus-infected culture supernatants were significantly higher in the upper anatomical location group than in the lower anatomical location group, after adjustment for patient-related characteristics (Figure 3A). The levels of IL-1β and IL-8 at 24 and 72 h p.i. were significantly higher in patients aged 21–64 years, whereas the levels of IL-6 were significantly higher in patients aged ≥ 65 years (Figure 3B). Male sex was significantly associated with higher levels of IL-1β, IL-6, IL-8, and IFN-γ at 24 h p.i., as well as IL-6, and IFN-γ at 72 h p.i. (Figure 3C). The presence of medical comorbidities was not significantly associated with cytokine levels at 24 and 72 h p.i.; however, changes in the IL-8 level were significantly associated with medical comorbidities after adjustment for anatomical location and other patient-related characteristics (Figure 3D). Except for a change in IL-8 level from 24 to 72 h p.i., obesity was not significantly associated with cytokine levels after adjustment, (Figure 3E).

## DISCUSSION

In line with the observations obtained in mouse and ferret models [5–7, 39], the H7N9 virus is not only able to penetrate the human respiratory epithelia [9], but also can successfully replicate in *ex vivo* tissues. However, neither the mouse nor the ferret model can mimic the severity of H7N9 infection in humans. In the present study, our objectives were to discover whether viral replication and cytokine responses of NHBE cells from two donors of different ages are distinct after H7N9 and H1N1pdm virus infection, and to validate whether these changes are different after H7N9 virus infection using primary epithelial cells from the respiratory tracts of 27 donors with various patient-related characteristics. In the second model, we used a primary culture approach to possibly retain the effects of patient-related characteristics. A standard operation procedure was used to culture all primary epithelial cells to maintain comparable culture conditions.

**Figure 4:**
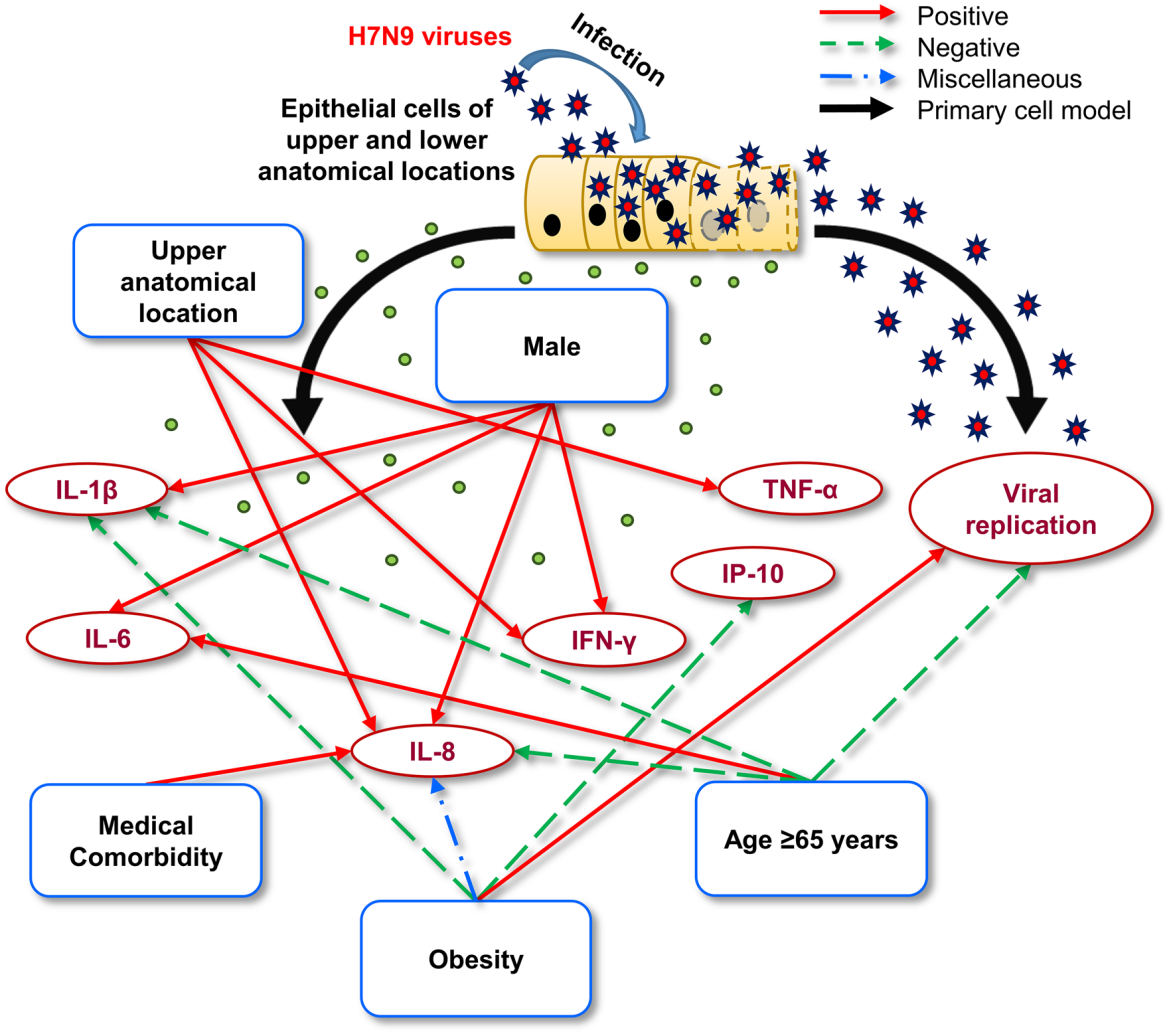
Schematic representation of the potential mechanisms by which different patient-related characteristics might influence the severity of infection Patient-related characteristics may influence: 1) The replication kinetics of the H7N9 virus; and 2) the individual cytokine response. The H7N9 virus can replicate equivalently in the epithelial cells obtained from the upper and lower anatomical locations, which may be negatively affected by age (≥ 65 years). After H7N9 infection, the anatomical location and patient-related characteristics are significantly positively, negatively, or miscellaneously associated with elevated levels of certain core cytokines.

We found that a donor's age might have an effect on viral RNA quantities (H7N9 and H1N1pdm) and cytokine levels (IL-1β, IL-8, IFN-γ, IP-10, and TNF-α) of the commercial NHBE culture supernatants, and certain patient-related characteristics could modulate viral replication and the cytokine response (e.g. age ≥ 65 years could decrease viral RNA quantity and the levels of IL-1β and IL-8, and increase the IL-6 level; male sex increased the levels of IL-1β, IL-6, IL-8, and IFN-γ; medical comorbidity increased the IL-8 level; and obesity increased the IL-8 level at 24 h p.i., and decreased the IL-8 level at 72 h p.i., increased the changes in viral RNA quantity, and decreased the changes in IL-1β and IP-10; Figure 4) for H7N9 infection in human respiratory tract primary epithelial cells. In addition, we also found that viral replication was equivalent in cells obtained from the upper and lower anatomical locations. Specifically, our data suggested that the level of IL-8 is markedly dependent on age, sex, medical comorbidities, and obesity.

In general, the levels of several cytokines were found to increase significantly from 24 to 72 h p.i. in NHBE and primary epithelial cell models (Table 1 & Table 3). Among them, IL-1β and IFN-γ, widely recognized as early markers of H7N9 infection [8, 18, 20], are involved in the modulation of the inflammatory response [40] and apoptosis [41]. High systemic levels of IFN-γ and IL-8 are associated with neutrophil activation [42] and can predict the need for inpatient care in H7N9-infected patients [43].

One of the main findings of this study was that the H7N9 virus showed equivalent and efficient replication in epithelial cells derived from both the upper and lower anatomical locations. This finding indicates that avian influenza A(H7N9) virus A/Taiwan/4/2014 [28], similar to avian influenza A(H7N9) virus A/Anhui/1/13 [9], can bind to both α2, 6-linked and α2, 3-linked sialic acids of the upper and lower respiratory tract epithelial cells. Intriguingly, infections of the upper airways were associated with a marked release of IL-8, IFN-γ, and TNF-α within 72 h p.i. These observations suggested that the H7N9 virus infection induces a site-specific, amplified, local innate immune response [44]. Notably, Chan *et al.* [22] found that H7N9 virus infection did not enhance the mRNA expression of IL-8 in peripheral blood monocyte-derived macrophages. These findings suggested that IL-8 released by H7N9-infected epithelial cells of the respiratory tract could be an important clue to the development of severe disease.

We demonstrated that the H7N9 virus replicated more slowly and induced lower levels of IL-1β and IL-8, accompanied by a more pronounced increase in IL-6 levels, in cell cultures obtained from patients aged > 65 years compared with those from patients aged 21–64 years. These observations may be attributable to “immunosenescence,” i.e. an age-related decline in immune function accompanied by the dysfunction of chemokine signaling pathways [45]. In contrast, younger subjects are more likely to survive after H7N9 infection [2, 10–14]. It is possible that the high viral replication occurring in their respiratory tract can act as a reservoir [46, 47]. By contrast, advanced age and increased IL-6 levels have been shown to be significant predictors of mortality [2, 8, 10–14, 18].

Male sex was significantly associated with higher levels of IL-1β, IL-6, IL-8, and IFN-γ between 24 and 72 h p.i. These findings might explain why men are at a higher risk of more severe disease. In our study, cells from patients with medical comorbidities produced higher IL-8 levels after experimental infection compared with those from otherwise healthy individuals, which might explain the absence of an association between medical comorbidities and the severity of H7N9 infections after adjustment for age and sex [14]. Obesity was significantly associated with a higher increase in viral RNA quantities; a higher level of IL-8 at 24 h and a lower level at 72 h p.i.; and less pronounced increases in IL-1β and IP-10. Our data indicated that cells derived from obese individuals have subtle but widespread changes in viral replication and cytokine response following experimental H7N9 infection. Furthermore, adiposity has been shown to be a critical factor in the age-associated lethal cytokine storm in an animal model [48]; nevertheless, the corresponding clinical effect might be modest.

Some limitations of our study merit comment. First, we used a primary epithelial culture model. Consequently, we cannot exclude the possibility that our findings related to H7N9 viral replication and its related cytokine response might be different *in vivo*. Second, because of the limited amount of primary epithelial cells, we did not include control virus infections in the primary culture model of explanted cells to verify the viral response. However, we confirmed that the H7N9 virus could replicate and induce a cytokine response differently from H1N1pdm virus in the NHBE experiment. Moreover, avian influenza A(H7N9) virus A/Anhui/1/13 could attach moderately or abundantly to both the upper and lower respiratory tract, and could be replicated in primary cultures of the nasopharynx and bronchus [9]. Nevertheless, the primary epithelial cells used in the present study seemed to reflect the properties of virus infection in patients, where the virus interacts with primary epithelial cells. Finally, the relatively small sample size precluded the analysis of a greater number of host characteristics that could affect virus replication kinetics and cytokine response. Further studies are needed to confirm and expand our findings.

In conclusion, we describe primary epithelial cellular models that might be useful to clarify the influence of patient-related characteristics on H7N9 viral replication and the associated cytokine response. Interestingly, IL-1β, IL-6, IL-8, IFN-γ, and TNF-α were the most differentially influenced by grouped host-specific features. Our results require independent replication in clinical studies before the widespread use of our primary cellular model can be recommended. However, our platform is less costly and labor-intensive than the use of animal models to mimic H7N9 virus infection. Our preliminary results using a primary cellular model have revealed many interesting differences among patient responses, and might represent a potential tool to identify individuals more prone to developing severe H7N9 infections.

## MATERIALS AND METHODS

### Ethics statement

Ethical approval for human tissue explants and the collection of demographic and clinical data was granted by the Institutional Review Board at the Chang Gung Medical Foundation, Taoyuan, Taiwan. (102-4819B). All the procedures described in the study complied with the principles of the Declaration of Helsinki and the standards of good clinical practice. Written informed consent was obtained from all participants. All experimental procedures were conducted in biosafety level 2 facilities by personnel wearing biosafety level 3 personal protective equipment.

### Participants

Between March 1, 2014 and April 30, 2016, we enrolled patients (*n* = 27) who were scheduled for upper or lower airway surgery at the Linkou Chang Gung Memorial Hospital (Taoyuan City, Taiwan). Inclusion criteria were as follows: 1) Age > 20 years; 2) need for upper airway surgery (for chronic hypertrophic rhinitis, chronic rhinosinusitis, or laryngeal cancer) or lung surgery (for lung cancer; with no prior history of radiation or chemotherapy); and 3) the existence of clinical records detailing the presence of medical comorbidities. Patients were excluded if they experienced respiratory tract viral infections within the two weeks preceding surgery, because some influenza viruses (such as H1N1) can trigger hypercytokinemia in the early stage and this effect can last for up to two weeks [20, 49]. Patients were also excluded if they had a known history of chronic viral infections (e.g., human immunodeficiency virus, hepatitis B virus, or hepatitis C virus), and/or were unwilling to participate.

### NHBE cells

NHBE cells from two human donors (24-year-old and 69-year-old female Caucasians) were purchased from Lonza (Lonza Walkersville, Inc., Walkersville, MD, USA) and maintained in bronchial epithelial growth media (Lonza Walkersville, Inc., Walkersville, MD, USA). Cells at the third passage were grown into a confluent monolayer for subsequent infection experiments.

### Primary cultures of human respiratory tract epithelial cells

Airway tissue specimens were collected from the following anatomical sites: 1) Inferior turbinate, 2) paranasal sinus, 3) larynx, and 4) bronchus. Immediately after harvesting, tissues were submerged in appropriate volumes of ice-cold Earl's balanced salt solution to preserve cell viability. Epithelial cells were expanded in parallel using standard methods [23–25]. After rinsing and removal of excess tissue, specimens were minced into cubes (2–3-mm^3^) and transferred onto a six-well Transwell cell culture system. Each Transwell was coated with collagen (30 μg/mL), fibronectin (10 μg/mL), and fetal bovine serum (10 μg/mL) and allowed to adhere. Culture medium (Dulbecco's Modified Eagle's Medium/Ham F12, 1% antibiotic/antimycotic, 1% fetal bovine serum) was added basolaterally to the co-culture dishes, and the plates were then incubated at 37 °C, with 5% CO_2_ in a humidified atmosphere. The culture solution was changed every two days. When the epithelial cells grew around the explant to cover a 1–2 cm^2^ area (approximately 2–3 weeks), tissue explants were transferred onto new scratched and coated plates. When confluent monolayers were observed, epithelial cells in the Transwells were infected with the H7N9 virus.

### Immunofluorescence staining

For fixation, 4% paraformaldehyde was applied to the cell slides at room temperature for 15 min, followed by three washes with phosphate-buffered saline (PBS). Cells were permeabilized with PBS containing 0.3% Triton X-100 (Sigma-Aldrich Co. LLC, Saint Louis, MO, USA) twice for 15 min at 4 °C. The cells were then incubated with 2% bovine serum albumin (Bionovas biotechnology Co., Ltd., Toronto, Ontario, Canada) in PBS for 30 min. Slides were then incubated at 37 °C for 1 h with anti-cytokeratin 19 antibodies (Abcam plc., Cambridge, UK) diluted 1:100 with blocking solution (final concentration 10 μg/mL), followed by three washes with PBS. Fluorescein isothiocyanate-conjugated AffiniPure goat anti-mouse IgG + IgM (H+L; Jackson ImmunoResearch Laboratories Inc., West Grove, PA, USA), diluted 1:100 to a final concentration of 15 μg/mL, was applied as the secondary antibody and incubated at 37 °C for 30 min, followed by three washes with PBS. For nuclear staining, slides were incubated with 4′, 6-diamidino-2-phenylindole (Life Technologies, Grand Island, NY, USA) for 15 min at room temperature, followed by three washes with PBS. Finally, the slide was dipped into PBS containing Evans blue for background counterstaining to show the localization of the cells.

### Viruses

We used influenza A/Taiwan/4-CGMH/2014 (TW4/H7N9) [28] and A/California/07/2009 (H1N1pdm) [27] in this study. All of the H7N9 infection-related procedures were conducted in biosafety level 3 facilities. A/Taiwan/4-CGMH/2014 is genetically close to the A/Anhui/1/2013 strain [28]. The viral RNA quantities were determined by a plaque assay on Madin–Darby canine kidney epithelial cells. Experimental infections of human respiratory tract primary epithelial cells (collected from different anatomical sites, as outlined above) were performed in triplicate. Cells (1 × 10^5^ cells/well) were initially exposed to serum-free bronchial epithelial cell basal medium (Lonza Walkersville, Inc.) containing 0.25% trypsin, and subsequently infected with the H7N9 virus at a multiplicity of infection of 0.01 at 37 °C, 5% CO_2_. Mock-infected cells served as a negative control. After infection, cells were washed five times with Hanks’ balanced salt solution to remove unbound viruses. At each predetermined time-point (see “Variables of interest”), virus-infected culture supernatants were collected and stored at −80°C until immediately before analysis.

### Viral RNA quantitation

All procedures were conducted in biosafety level 2 facilities by personnel wearing biosafety level 3 personal protective equipment. Quantitative real-time reverse transcription polymerase chain reaction (qRT-PCR) was used for H7N9 virus load quantitation. Viral RNA was extracted from culture supernatants using a QIAamp viral RNA mini kit (Qiagen Inc., Valencia, CA, USA) and subjected to qRT-PCR. We used the FluA primers and probe originally developed by the World Health Organization Collaborating Centre in Beijing, China (2013). The nucleotide sequences were as follows: forward primer, 5′-GACCRATCCTGTCACCTCTGAC-3′; reverse primer, 5′- AGGGCATTYTGGACAAAKCGTCTA-3′; probe, 5′-FAM-TGCAGTCCTCGCTCACTGGGCACG-BHQ1-3′. In brief, 140 μL of supernatant was mixed with 560 μL of lysis buffer (350 μL for up to 5 × 10^6^ cells). The volume of extracted RNA was adjusted to 30 μL with RNase-free water. Negative controls consisted of sterile water and were subjected to all the preparation steps in parallel with the extracted samples. A RealTime ready RNA virus master kit (Roche Diagnostics, GmbH Mannheim, Germany) was used for amplification. To set up the amplification reaction, 5 μL of template was added to each reaction tube; the working concentrations for the primers and probe were 10 and 5 μM, respectively. The final volume for the qRT-PCR was 25 μL. Reverse transcription and amplification were carried out in a one-step reaction on a Bio-Rad PCR system (CFX96; Bio-Rad Laboratories, Hercules, CA, USA). The conditions for qRT-PCR were as follows: 45 °C for 10 min, 95 °C for 10 min; followed by 40 cycles at 95 °C for 15 s and 60 °C for 45 s. The qRT-PCR was repeated for samples with a Ct value > 38. PCR products were cloned into a plasmid to generate positive controls. For quantification, plasmid DNAs at six different concentrations, from 10 copies/μL to 10^6^ copies/μL, were run in parallel with all the samples.

### Quantification of cytokines in virus-infected culture supernatants

The concentrations of six core cytokines in virus-infected culture supernatants were determined using the Bio-Plex Pro Human Cytokine 27-plex panel (Bio-Rad Laboratories). Specifically, the following analytes were included for statistical analysis: IL-1β, IL-6, IL-8, IFN-γ, IP-10, and TNF-α. Samples were initially incubated on antibody-coupled beads for 60 min to allow binding, followed by incubation with detection antibodies for 30 min. Conjugates were treated with streptavidin for 10 min, washed on a Bio-Plex Pro II wash station, resuspended, vortexed, and quantified by fluorescence. All data were analyzed using a Bio-Rad Bio-Plex Luminex 200 instrument. Standard curves (Log (x) – Linear (y)) were generated with the Bio-Rad Bio-Plex Manager v6.0 software. Triplicate measurements of all samples were performed.

### Variables of interest

Viral RNA quantities and cytokine levels in the H7N9-infected culture supernatants at 24 h and 72 h p.i. served as the main variables of interest.

### Sample size

The differences in viral RNA quantities and cytokine levels at 24 h p.i. were expected to be less significant than those at 72 h p.i.; therefore, a more conservative power analysis was performed, based on the variables of interest at 24 h p.i. The priori sample size of the model of primary cultures of human respiratory tract epithelial cells was estimated using outcome effects (viral RNA quantities and cytokine levels) based on a pilot study of the model of NHBE cells (mean 4.95 [standard deviation 0.94] and mean 3.61 [standard deviation 0.34] for “aged 24 years” and “aged 69 years”, respectively), and a two-tailed Mann–Whitney *U* test. To reach 90% power with an effect size of 1.9, a type I error of 0.05, and an allocation ratio 0.2, we obtained a minimum sample size of 26 (22 and four patients for “aged 21–64 years” and “aged ≥ 65 years”, respectively) for comparison of IP-10 levels at 24 h p.i.

### Statistical analysis

Viral RNA quantities and cytokine levels were log-transformed (log_10_) to improve the normality of the distribution. To avoid gaps in the assay results and inaccuracies in the estimates of concentrations, a value of 0.1 pg/mL was entered in the data set when an analyte was undetectable [20]. Means and standard errors of the mean were used to summarize continuous variables. The viral RNA quantities and cytokines were compared using the Wilcoxon signed rank test, the Kruskal–Wallis test, or the Mann–Whitney *U* test, as appropriate. At selected time-points, viral RNA quantities and cytokine levels were compared according to both the anatomical locations of the explant and patient-related characteristics of interest using GEE models [36], which were employed to determine whether changes in virus and cytokine levels were significantly different at specific time-points after adjusting for anatomical location and patient-related characteristics, or between different anatomical locations and patient-related characteristics after adjustment for other confounding variables. The use of GEE allowed the monitoring of viral RNA quantities and cytokine level as a function of time, anatomical location, age, sex, medical comorbidities, and obesity. The anatomical locations of the explants were dichotomized for the purpose of analysis in the upper (inferior turbinate and paranasal sinus) *versus* lower (larynx and bronchus) respiratory tract. Spearman's rank correlation coefficients were used to analyze the associations between patient-related characteristics, viral RNA quantities, and cytokine levels. We applied Benjamini and Hochberg's approach to control the false discovery rate. All statistical calculations were performed with the G^*^Power 3.1.9.2 software (Heinrich-Heine University, Dusseldorf, Germany), the IBM SPSS (version 23; International Business Machines Corp.), and the GraphPad Prism for Windows (version 7.0; GraphPad Software, Inc.) software packages. A *P* value < 0.05 (two-tailed) was considered statistically significant.

### Ethical approval

The study was approved by the Institutional Review Board at the Chang Gung Medical Foundation, Taoyuan, Taiwan. (102-4819B, 104-9560C).

## Abbreviations

GEE: generalized estimating equation
IFN: interferon
IL: interleukin
IP-10: Interferon-γ-induced protein 10
NHBE: normal human bronchial epithelial
PBS: phosphate-buffered saline
PCR: polymerase chain reaction
p.i.: post infection
qRT-PCR: quantitative real-time reverse transcription polymerase chain reaction
TNF: tumor necrosis factor

## References

[1] Gao R, Cao B, Hu Y, Feng Z, Wang D, Hu W, Chen J, Jie Z, Qiu H, Xu K, Xu X, Lu H, Zhu W (2013). Human infection with a novel avian-origin influenza A (H7N9) virus. N Engl J Med.

[2] Gao HN, Lu HZ, Cao B, Du B, Shang H, Gan JH, Lu SH, Yang YD, Fang Q, Shen YZ, Xi XM, Gu Q, Zhou XM (2013). Clinical findings in 111 cases of influenza A (H7N9) virus infection. N Engl J Med.

[3] Zhou L, Ren R, Yang L, Bao C, Wu J, Wang D, Li C, Xiang N, Wang Y, Li D, Sui H, Shu Y, Feng Z (2017). Sudden increase in human infection with avian influenza A(H7N9) virus in China, September-December 2016. Western Pac Surveill Response J.

[4] Yu H, Wu JT, Cowling BJ, Liao Q, Fang VJ, Zhou S, Wu P, Zhou H, Lau EHY, Guo D, Ni MY, Peng Z, Feng L (2014). Effect of closure of live poultry markets on poultry-to-person transmission of avian influenza A H7N9 virus: an ecological study. The Lancet.

[5] Zhang Q, Shi J, Deng G, Guo J, Zeng X, He X, Kong H, Gu C, Li X, Liu J, Wang G, Chen Y, Liu L (2013). H7N9 influenza viruses are transmissible in ferrets by respiratory droplet. Science.

[6] Xu L, Bao L, Deng W, Zhu H, Chen T, Lv Q, Li F, Yuan J, Xiang Z, Gao K, Xu Y, Huang L, Li Y (2013). The mouse and ferret models for studying the novel avian-origin human influenza A (H7N9) virus. Virol J.

[7] Richard M, Schrauwen EJ, de Graaf M, Bestebroer TM, Spronken MI, van Boheemen S, de Meulder D, Lexmond P, Linster M, Herfst S, Smith DJ, van den Brand JM, Burke DF (2013). Limited airborne transmission of H7N9 influenza A virus between ferrets. Nature.

[8] Zhou J, Wang D, Gao R, Zhao B, Song J, Qi X, Zhang Y, Shi Y, Yang L, Zhu W, Bai T, Qin K, Lan Y (2013). Biological features of novel avian influenza A (H7N9) virus. Nature.

[9] van Riel D, Leijten LM, de Graaf M, Siegers JY, Short KR, Spronken MI, Schrauwen EJ, Fouchier RA, Osterhaus AD, Kuiken T (2013). Novel avian-origin influenza A (H7N9) virus attaches to epithelium in both upper and lower respiratory tract of humans. Am J Pathol.

[10] Knepper J, Schierhorn KL, Becher A, Budt M, Tonnies M, Bauer TT, Schneider P, Neudecker J, Ruckert JC, Gruber AD, Suttorp N, Schweiger B, Hippenstiel S (2013). The novel human influenza A(H7N9) virus is naturally adapted to efficient growth in human lung tissue. MBio.

[11] Arima Y, Vong S, World Health Organization Outbreak Response Team (2013). Human infections with avian influenza A(H7N9) virus in China: preliminary assessments of the age and sex distribution. Western Pac Surveill Response J.

[12] Chen X, Yang Z, Lu Y, Xu Q, Wang Q, Chen L (2013). Clinical features and factors associated with outcomes of patients infected with a Novel Influenza A (H7N9) virus: a preliminary study. PLoS One.

[13] Liu B, Havers F, Chen E, Yuan Z, Yuan H, Ou J, Shang M, Kang K, Liao K, Liu F, Li D, Ding H, Zhou L (2014). Risk factors for influenza A(H7N9) disease--China, 2013. Clin Infect Dis.

[14] Xiao YY, Cai J, Wang XY, Li FD, Shang XP, Wang XX, Lin JF, He F (2015). Prognosis and survival of 128 patients with severe avian influenza A(H7N9) infection in Zhejiang province, China. Epidemiol Infect.

[15] Huo X, Xu K, Dai Q, Qi X, Yu H, Bao C (2015). Age and gender adjusted comparison of clinical features between severe cases infected with H7N9 and H1N1pdm influenza A in Jiangsu Province, China. PLoS One.

[16] Jane-Wit D, Chun HJ (2012). Mechanisms of dysfunction in senescent pulmonary endothelium. J Gerontol A Biol Sci Med Sci.

[17] Shen Z, Chen Z, Li X, Xu L, Guan W, Cao Y, Hu Y, Zhang J (2014). Host immunological response and factors associated with clinical outcome in patients with the novel influenza A H7N9 infection. Clin Microbiol Infect.

[18] Wang Z, Zhang A, Wan Y, Liu X, Qiu C, Xi X, Ren Y, Wang J, Dong Y, Bao M, Li L, Zhou M, Yuan S (2014). Early hypercytokinemia is associated with interferon-induced transmembrane protein-3 dysfunction and predictive of fatal H7N9 infection. Proc Natl Acad Sci U S A.

[19] Yang ZF, Mok CK, Liu XQ, Li XB, He JF, Guan WD, Xu YH, Pan WQ, Chen LY, Lin YP, Wu SG, Pan SH, Huang JC (2015). Clinical, virological and immunological features from patients infected with re-emergent avian-origin human H7N9 influenza disease of varying severity in Guangdong province. PLoS One.

[20] Guo J, Huang F, Liu J, Chen Y, Wang W, Cao B, Zou Z, Liu S, Pan J, Bao C, Zeng M, Xiao H, Gao H (2015). The serum profile of hypercytokinemia factors identified in H7N9-infected patients can predict fatal outcomes. Sci Rep.

[21] Wu JT, Ma ES, Lee CK, Chu DK, Ho PL, Shen AL, Ho A, Hung IF, Riley S, Ho LM, Lin CK, Tsang T, Lo SV (2010). The infection attack rate and severity of 2009 pandemic H1N1 influenza in Hong Kong. Clin Infect Dis.

[22] Chan MC, Chan RW, Chan LL, Mok CK, Hui KP, Fong JH, Tao KP, Poon LL, Nicholls JM, Guan Y, Peiris JS (2013). Tropism and innate host responses of a novel avian influenza A H7N9 virus: an analysis of *ex-vivo* and *in-vitro* cultures of the human respiratory tract. Lancet Respir Med.

[23] Yaghi A, Zaman A, Dolovich M (2010). Primary human bronchial epithelial cells grown from explants. J Vis Exp.

[24] Bochkov YA, Palmenberg AC, Lee WM, Rathe JA, Amineva SP, Sun X, Pasic TR, Jarjour NN, Liggett SB, Gern JE (2011). Molecular modeling, organ culture and reverse genetics for a newly identified human rhinovirus C. Nat Med.

[25] Guo-Parke H, Canning P, Douglas I, Villenave R, Heaney LG, Coyle PV, Lyons JD, Shields MD, Power UF (2013). Relative respiratory syncytial virus cytopathogenesis in upper and lower respiratory tract epithelium. Am J Respir Crit Care Med.

[26] Mindaye ST, Ilyushina NA, Fantoni G, Alterman MA, Donnelly RP, Eichelberger MC (2017). Impact of influenza A virus infection on the proteomes of human bronchoepithelial cells from different donors. J Proteome Res.

[27] Kiseleva I, Larionova N, Kuznetsov V, Rudenko L (2010). Phenotypic characteristics of novel swine-origin influenza A/California/07/2009 (H1N1) virus. Influenza Other Respir Viruses.

[28] Chen GW, Kuo SM, Yang SL, Gong YN, Hsiao MR, Liu YC, Shih SR, Tsao KC (2016). Genomic signatures for avian H7N9 viruses adapting to humans. PLoS One.

[29] Tisoncik JR, Korth MJ, Simmons CP, Farrar J, Martin TR, Katze MG (2012). Into the eye of the cytokine storm. Microbiol Mol Biol Rev.

[30] Khair OA, Devalia JL, Abdelaziz MM, Sapsford RJ, Tarraf H, Davies RJ (1994). Effect of Haemophilus influenzae endotoxin on the synthesis of IL-6, IL-8, TNF-alpha and expression of ICAM-1 in cultured human bronchial epithelial cells. Eur Respir J.

[31] Mills PR, Davies RJ, Devalia JL (1999). Airway epithelial cells, cytokines, and pollutants. Am J Respir Crit Care Med.

[32] Maurer M, von Stebut E (2004). Macrophage inflammatory protein-1. Int J Biochem Cell Biol.

[33] Lewandowska-Polak A, Brauncajs M, Paradowska E, Jarzebska M, Kurowski M, Moskwa S, Lesnikowski ZJ, Kowalski ML (2015). Human parainfluenza virus type 3 (HPIV3) induces production of IFNgamma and RANTES in human nasal epithelial cells (HNECs). J Inflamm (Lond).

[34] Rajan D, McCracken CE, Kopleman HB, Kyu SY, Lee FE, Lu X, Anderson LJ (2014). Human rhinovirus induced cytokine/chemokine responses in human airway epithelial and immune cells. PLoS One.

[35] Pan WH, Lee MS, Chuang SY, Lin YC, Fu ML (2008). Obesity pandemic, correlated factors and guidelines to define, screen and manage obesity in Taiwan. Obes Rev.

[36] Liang KY, Zeger SL (1986). Longitudinal data analysis using generalized linear models. Biometrika.

[37] Chi Y, Zhu Y, Wen T, Cui L, Ge Y, Jiao Y, Wu T, Ge A, Ji H, Xu K, Bao C, Zhu Z, Qi X (2013). Cytokine and chemokine levels in patients infected with the novel avian influenza A (H7N9) virus in China. J Infect Dis.

[38] Jiang Y, Xu J, Zhou C, Wu Z, Zhong S, Liu J, Luo W, Chen T, Qin Q, Deng P (2005). Characterization of cytokine/chemokine profiles of severe acute respiratory syndrome. Am J Respir Crit Care Med.

[39] Meliopoulos VA, Karlsson EA, Kercher L, Cline T, Freiden P, Duan S, Vogel P, Webby RJ, Guan Y, Peiris M, Thomas PG, Schultz-Cherry S (2014). Human H7N9 and H5N1 influenza viruses differ in induction of cytokines and tissue tropism. J Virol.

[40] Netea MG, Kullberg BJ, Van der Meer JW (2000). Circulating cytokines as mediators of fever. Clin Infect Dis.

[41] Brydon EW, Morris SJ, Sweet C (2005). Role of apoptosis and cytokines in influenza virus morbidity. FEMS Microbiol Rev.

[42] Zeng H, Belser JA, Goldsmith CS, Gustin KM, Veguilla V, Katz JM, Tumpey TM (2015). A(H7N9) virus results in early induction of proinflammatory cytokine responses in both human lung epithelial and endothelial cells and shows increased human adaptation compared with avian H5N1 virus. J Virol.

[43] Bermejo-Martin JF, Ortiz de Lejarazu R, Pumarola T, Rello J, Almansa R, Ramirez P, Martin-Loeches I, Varillas D, Gallegos MC, Seron C, Micheloud D, Gomez JM, Tenorio-Abreu A (2009). Th1 and Th17 hypercytokinemia as early host response signature in severe pandemic influenza. Crit Care.

[44] Herold S, Becker C, Ridge KM, Budinger GR (2015). Influenza virus-induced lung injury: pathogenesis and implications for treatment. Eur Respir J.

[45] Kennedy RB, Ovsyannikova IG, Haralambieva IH, Oberg AL, Zimmermann MT, Grill DE, Poland GA (2016). Immunosenescence-related transcriptomic and immunologic changes in older individuals following influenza vaccination. Front Immunol.

[46] Nishiura H, Mizumoto K, Ejima K (2013). How to interpret the transmissibility of novel influenza A(H7N9): an analysis of initial epidemiological data of human cases from China. Theor Biol Med Model.

[47] Short KR, Kroeze EJ, Fouchier RA, Kuiken T (2014). Pathogenesis of influenza-induced acute respiratory distress syndrome. Lancet Infect Dis.

[48] Mirsoian A, Bouchlaka MN, Sckisel GD, Chen M, Pai CC, Maverakis E, Spencer RG, Fishbein KW, Siddiqui S, Monjazeb AM, Martin B, Maudsley S, Hesdorffer C (2014). Adiposity induces lethal cytokine storm after systemic administration of stimulatory immunotherapy regimens in aged mice. J Exp Med.

[49] Almansa R, Anton A, Ramirez P, Martin-Loeches I, Banner D, Pumarola T, Xu L, Blanco J, Ran L, Lopez-Campos G, Martin-Sanchez F, Socias L, Loza A (2011). Direct association between pharyngeal viral secretion and host cytokine response in severe pandemic influenza. BMC Infect Dis.

